# GTSE1-expressed osteoblastic cells facilitate formation of pro-metastatic tumor microenvironment in osteosarcoma

**DOI:** 10.1016/j.gendis.2025.101591

**Published:** 2025-03-07

**Authors:** Linzhu Wang, Wenyue Li, Weihang Ji, Danyang Bing, Mingyue Liu, Kaidong Liu, Bo Chen, Zhangxiang Zhao, Yunyan Gu, Xuelian Li, Xiaoqiang E, Lei Yang

**Affiliations:** a Department of Pharmacology (State-Province Key Laboratories of Biomedicine-Pharmaceutics of China, Key Laboratory of Cardiovascular Research, Ministry of Education), State Key Laboratory of Frigid Zone Cardiovascular Diseases (SKLFZCD), College of Pharmacy, Harbin Medical University, Harbin, Heilongjiang 150081, China; b Department of Systems Biology, College of Bioinformatics Science and Technology, Harbin Medical University, Harbin, Heilongjiang 150081, China; c Clinical Research Center (CRC), Medical Pathology Center (MPC), Cancer Early Detection and Treatment Center (CEDTC), Chongqing University Three Gorges Hospital, Chongqing University, Wanzhou, Chongqing 404100, China; d Department of Orthopedics, The First Affiliated Hospital of Harbin Medical University, Harbin, Heilongjiang 150081, China; e Key Laboratory of Hepatosplenic Surgery of Ministry of Education, The First Affiliated Hospital of Harbin Medical University, Harbin, Heilongjiang 150081, China; f NHC Key Laboratory of Cell Transplantation, The First Affiliated Hospital of Harbin Medical University, Harbin, Heilongjiang 150081, China; g Department of Breast Surgery, Harbin Medical University Cancer Hospital, Harbin, Heilongjiang 150081, China

**Keywords:** *GTSE1*, Metastasis, Osteosarcoma, Single-cell transcriptome, Tumor microenvironment

## Abstract

Understanding metastatic osteosarcoma relies on defining the complexity of cell types, their associated molecular profiles, and interactions among cells in the tumor microenvironment. Here, we integrated single-cell and bulk gene expression datasets and revealed that metastatic lesions were highly enriched for *GTSE1*^+^ osteoblasts (OB). Under the regulation of E2F family members, *GTSE1*^+^ OB cells harbored enhanced proliferation activity and high differentiation potential. Augmentation of *GTSE1* enhanced the abilities of cell migration and invasion, while silencing of *GTSE1* impaired the abilities in human OB cell lines. Furthermore, cellular communication analysis showed the cross-talk between *GTSE1*^+^ OB cells and CD8^+^ T cells in metastasis was achieved through the *MIF*-(*CD74*-*CXCR4*) pair. Spatial transcriptomic data revealed that *MIF*-*CD74* and *CXCR4*-*MIF*/*CD74* showed a higher positive correlation in undifferentiated pleomorphic sarcoma than leiomyosarcoma. Correlation analysis unveiled that *GTSE1*^+^ OB cells and monocytes were the negatively correlated populations at the single-cell level, a finding validated in 4 independent osteosarcoma datasets comprising 226 samples. Our findings suggest that *GTSE1* overexpression serves as a potential biomarker for metastasis in osteosarcoma and provides a promising strategy to prevent metastasis by targeting *GTSE1*^+^ OB cells.

## Introduction

Osteosarcoma (OS), notorious for its poor prognosis, stands as one of the most malignant tumors in bone.^1^ Remarkably, approximately 40%–50% of the patients with OS have clinically detectable metastasis, requiring the use of intensive adjuvant chemotherapy.^2^ Metastasis causes a considerable obstacle to the long-term survival of patients with OS, with a five-year survival rate of less than 20%.^3^ Defining the cellular and molecular underlying diversity is key to understanding the progression from primary to metastatic OS.

Cancer metastasis, a greatly intricate process, is closely correlated with tumor microenvironment.^4^ Recently, tumor microenvironment gained significance besides its conventional role of cellular support as a genuine contributor to cancer progression and metastasis.^5^ To date, a handful of studies estimated the immune infiltration in OS.^6^^,^^7^ While these studies provided valuable insights, they relied on bulk expression level, ignoring gene expression patterns at the single-cell level.

Single-cell RNA sequencing (scRNA-seq) showed beneficial values in providing biological insights into a variety of cancers, including OS.^8^ A study revealed that the metastatic microenvironment underwent reprogramming of *MRC1*^+^ *CCL18*^+^ M2-like macrophages in colorectal cancer based on scRNA-seq data.^9^ Previous single-cell transcriptome studies of OS focused either on intratumoral heterogeneity or on the construction of prognosis models,^8^^,^^10^ but did not clarify the effects and mechanisms of specific cells in OS metastasis. Cancer-associated fibroblasts, characterized by significant overexpression of *LOX*, were found with a high infiltration proportion in recurrent OS, leading to epithelial–mesenchymal transition and poor prognosis of OS.^11^ Another study found that the *CD24*^+^ cell subset facilitated invasion and metastasis of OS,^12^ yet little was known about the expression distribution of *CD24* at the single-cell level. Less clear is whether specific cell subsets present in OS may drive metastasis.

By analyzing single-cell and bulk gene expression datasets from primary and metastatic samples, we mapped the cell atlas of OS. We characterized diverse cell composition patterns in the tumor microenvironment between primary and metastatic lesions. We also revealed the features of metastasis-enrich cells, including their gene expression patterns, molecular signatures, transcriptional regulation, as well as developmental trajectories. *In vitro* assays, consistent with bioinformatics analysis, proved that *GTSE1* up-regulation promoted the invasion and migration of osteoblasts (OB). Our findings provided new insights into the biology of OS metastasis and uncovered *GTSE1* as a potential promoter of metastasis warranting further investigation.

## Materials and methods

### Datasets

Table S1 showed the statistics of OS samples. For bulk RNA expression profiles, annotation files supplied from the matching detection platforms were used to convert probes to gene level. If a probe did not map to any Gene ID or map to multiple Gene IDs, it was deleted. If multiple probe sets were mapped to the same gene, the averaging value represented its expression value.

### Processing of the scRNA-seq data

The R package “Seurat” (v 4.2.1) was used to process data of single cells.^13^ Cells with more than 300 detected genes and a percentage of mitochondrial genes below 10% of total expressed genes were retained; genes detected in fewer than 3 cells were filtered out. We removed potential doublets in each sample using the R package “DoubletFinder” (v 2.0.3).^14^ A total of 85,491 qualified cells were used for downstream analysis. FindIntegrationAnchors and IntegrateData functions of Seurat were used to integrate scRNA-seq data from nine samples. Subsequently, count data was normalized using Seurat's NormalizeData function. The scaled expression data for the top 3000 highly variable genes (identified using the FindVariableFeatures function of Seurat) served as input for principal component analysis. We performed the FindNeighbors function to identify nearest neighbors for graph clustering based on the top 30 principal components and performed the FindCluster function to gain cell subsets. t-distributed stochastic neighbor embedding (t-SNE) algorithm was used to visualize identified clusters on a 2D map. Cell types were annotated based on the canonical marker genes derived from the literature or the CellMarker database.^8^^,^^15^

### Cellular proportion analysis

The scDC algorithm was used to perform the bias-corrected and accelerated bootstrap analysis with the function scDC_noClustering (cellTypes, subject, calCI = TRUE, calCI_method = BCa, nboot = 10,000).^16^ Median value of bootstrap resampled cellular composition was selected to chase down the metastasis-enrich cellular subsets.

### Cell-specific DEG identification and pathway enrichment analysis

The FindMarkers function of Seurat was employed to identify differential expression genes (DEGs) of each subset (false discovery rate < 0.05, only.pos = T and logfc.threshold = 0.1). Firstly, we acquired the differentially overrepresented genes of one specified subset compared with each of the others. Secondly, we retained consistently differentially overrepresented genes as one subset of cell-specific DEGs. The same parameters were set for identifying the differentially overrepresented genes in metastatic subsets compared with primary subsets. Subsequently, the subset-specific Gene Ontology (GO) biological process was computed by the “clusterProfiler” (v 4.0.5) and “DOSE” (v 3.18.3) packages of R and the metastasis-specific GO biological process was calculated with the compareCluster function of Seurat.

The curated gene sets of G2M and E2F targets (*e.g.*, pathways) were obtained from MSigDB.^17^ The sample-wise gene set enrichment score for each cell or sample was assessed using single-sample gene set enrichment analysis (ssGSEA). The proliferation scores of cancer cells were estimated based on the average normalized expression of signature genes (*MKI67*, *IGF1*, *ITGB2*, *PDGFC*, *JAG1*, and *PHGDH*).^18^ We used the two-tailed Wilcoxon rank-sum test to compare the enrichment scores between two subsets. Cell cycle scoring was calculated based on the expression of cell cycle phase marker genes using the CellCycleScoring function of Seurat.

### Survival analysis and Scissor analysis

See Supplementary Methods for a detailed preprocess.

### Cell culture and transfection

See Supplementary Methods for a detailed preprocess.

### Quantitative real-time PCR

See Supplementary Methods for a detailed preprocess.

### Western blotting

See Supplementary Methods for a detailed preprocess.

### CCK-8 assay

Cell viability was detected after 24 h transfection using a CCK-8 kit (Meilun Biotechnology, Dalian, China) according to the manufacturer's instructions. Data were reproduced at least six times in independent experiments.

### Wound healing assay

The OB cells were plated into 6-well culture plates. When the confluence of cells reached 60%, the cells were transfected with siRNA or plasmid. Then, the cell monolayer was gently scratched with a 200 μL pipette tip. The cells were washed with phosphate buffer saline solution and renewed serum-free medium. The images were taken by the Nikon Ts100 microscope (Nikon, Tokyo, Japan) at 0 and 24 h. The wound area was analyzed using Image J software. Migration rate (%) = 1 − (24 h scratch area/0 h scratch area) × 100%.

### Transwell assay

The OB cells transfected with siRNA or plasmid were cultured at 1 × 10^4^ cells/200 μL serum-free medium in each Transwell® cell culture insert (8-μm pore size, 6.5-mm diameter; Costar, Cambridge, MA, USA). The medium containing 10% fetal bovine serum was added to the lower chamber. After 48 h, the filters were washed with phosphate buffer saline solution, fixed with methanol for 10 min, and stained with 0.1% crystal violet (Beyotime, Jiangsu, China) for 15 min. Invasion cells were counted under the microscope.

### Colony formation assay

The OB cells transfected with siRNA or plasmid were seeded in 6-well culture plates with a concentration of 500 cells per well. After 14 days, cells were fixed with methanol for 10 min and stained with 0.1% crystal violet for 15 min. Colonies were air-dried and counted. The experiments were repeated three times.

### Correlation analysis

See Supplementary Methods for a detailed preprocess.

### Estimation of transcription factor activity

Human regulons (grades A–C) were obtained from the R package “DoRothEA” (v 1.10.0) and differential analysis was conducted on scaled VIPER scores among subsets, based on the expression matrix.^19^ Differences in transcription factors among subsets were estimated using the DoRothEA algorithm.^20^ Transcription factors were ranked according to the fold change of their corresponding VIPER scores and the top 20 highly variable scores were retained for heatmap visualization.

### Pseudotime trajectory analysis

Pseudo-temporal trajectory analysis was performed using the R package Monocle 3.0 (v 1.0.0).^21^ We adapted the cancer cells' Seurat object to generate a Monocle3 object by the new_cell_data_set function. The dimensionality of the data was reduced using uniform manifold approximation and projection (UMAP) with the reduce_dimension function. The trajectory graph of cancer cells was inferred by the learn_graph function with the default parameters.

### Intercellular ligand–receptor interaction inference

Inferring the ligand-receptor communication between subsets using the R package “CellPhoneDB” (v 3.0.2).^22^ As for the differential cross-talk analysis (metastatic OS *vs*. primary OS) between cancer cells and non-malignant cells, we calculated the cross-talk between cancer cells and non-malignant cells in primary OS and metastatic OS, respectively. Ligand–receptor pairs exhibiting significant cross-talk only in metastasis were selected, and R package “circlize” (v 0.4.15) and “ComplexHeatmap” (v 2.8.0) were used to visualize the results. Furthermore, the R package “CellChat” (v 1.1.3) was applied to analyze the cell communication between *GTSE1*^+^ OB cells and CD8^+^ T cells.^23^

### Spatial transcriptomic data processing and analysis

Processed spatial transcriptomics count matrices for three samples from GSE212526 were loaded into the R package “Seurat”. We defined the colocalization of *MIF*-*CD74*, *CXCR4*-*MIF*, and *CXCR4*-*CD74* pairs as the geometric mean of expression values of each pair. Pearson correlation analysis was computed across spots between the co-expression of *MIF*-*CD74* and *CXCR4*-*MIF*/*CD74*.

### Statistical analysis

All bioinformatics analyses in this study were performed using R software (v 4.1.1, http://www.r-project.org/). Experimental statistical analyses were performed using GraphPad Prism software (v 9.0, https://www.graphpad.com/scientific-software/prism/). Data were presented as mean ± standard deviation and statistical significance was calculated using a two-tailed student's *t*-test. *p* < 0.05 was considered statistically significant (∗*p* < 0.05, ∗∗*p* < 0.01, ∗∗∗*p* < 0.001). “ns” indicates no significance.

## Results

### Single-cell RNA transcriptome reveals heterogeneity between primary and metastatic OS

**Figure 1:**
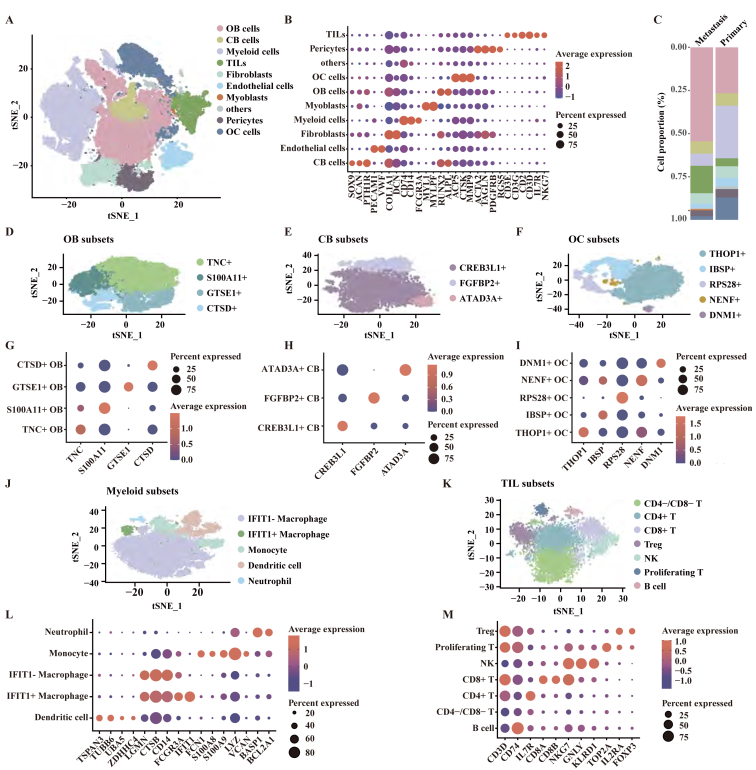
Single-cell RNA transcriptome revealed heterogeneity between primary and metastatic OS. **(A)** t-SNE visualization of major cell lineages. **(B)** The dot plots showing the average expression of 22 signature genes across the 10 cellular clusters. The dot sizes indicated the proportions of cells expressing the genes in each cluster and the color spectrum represented the mean expression levels of the signature genes. **(C)** Relative proportions of 10 clusters across primary and metastatic tissues as indicated. **(D**–**F)** t-SNE visualization of OB cell subsets (D), CB cell subsets (E), and OC cell subsets (F). **(G**–**I)** The dot plots showing the average expression of selected signature genes in OB cell subsets (G), CB cell subsets (H), and OC cell subsets (I). The dot sizes indicated the proportions of cells expressing the genes in each subset and the color spectrum represented the mean expression levels of the markers. **(J, K)** t-SNE visualization of myeloid cell subsets (J), and TIL subsets (K). **(L, M)** Dot plots showing average expression of selected signature genes in myeloid cell subsets (L), and TIL subsets (M). The dot sizes indicated the proportions of cells expressing the genes in each subset and the color spectrum represented the mean expression levels of the markers.

A scRNA-seq dataset comprising seven primary OS samples and two metastatic OS samples was collected to elucidate the cellular composition (Fig. S1A). After quality-control filtering, gene expression profiles of 67,265 cells from primary patients and 18,226 cells from metastatic patients were retained for further analyses. Unsupervised graph-based clustering of cells after dimensionality reduction identified several clusters, which could be assigned to OB cells, tumor-infiltrating lymphocytes (TILs), chondroblasts (CB), fibroblasts, endothelial cells, myeloid cells, osteoclasts (OC), pericytes, myoblasts, and a cluster named others (Fig. 1A, B). t-SNE visualization showed a well-mixed distribution of cells in the integrated data (Fig. S1B). Metastatic samples were enriched for OB cells and TILs while containing fewer myeloid cells and OC cells than primary samples (Fig. 1C), suggesting the intertumoral heterogeneity among lesions.

The histological subtypes of scRNA-seq samples were OB and CB, and five clusters were extracted using Seurat to further define cell subsets, including OB cells, TILs, myeloid cells, OC cells, and CB cells. The OB lineage subsets were characterized by relatively high expression of *TNC*, *S100A11*, *GTSE1*, and *CTSD* (Fig. 1D, G), while the CB lineage subsets were identified based on relatively high expression of *CREB3L1*, *FGFBP2*, and *ATAD3A* (Fig. 1E, H). The OC cells were divided into five subsets with relatively high expression of *THOP1*, *IBSP*, *RPS28*, *NENF*, and *DNM1* (Fig. 1F, I). Myeloid cells were clustered into neutrophils, monocytes, dendritic cells, *IFIT1*^+^ macrophages, and *IFIT1*^-^ macrophages (Fig. 1J, L). TILs were classified into seven subsets, regulatory T (Treg) cells, proliferating T cells, natural killer (NK) cells, CD8^+^ T cells, CD4^+^ T cells, CD4^−^/CD8^−^ T cells, and B cells (Fig. 1K, M; Fig. S1C).

### *GTSE1*^+^ OB cells and *CREB3L1*^+^ CB cells are enriched in metastatic tumors

**Figure 2:**
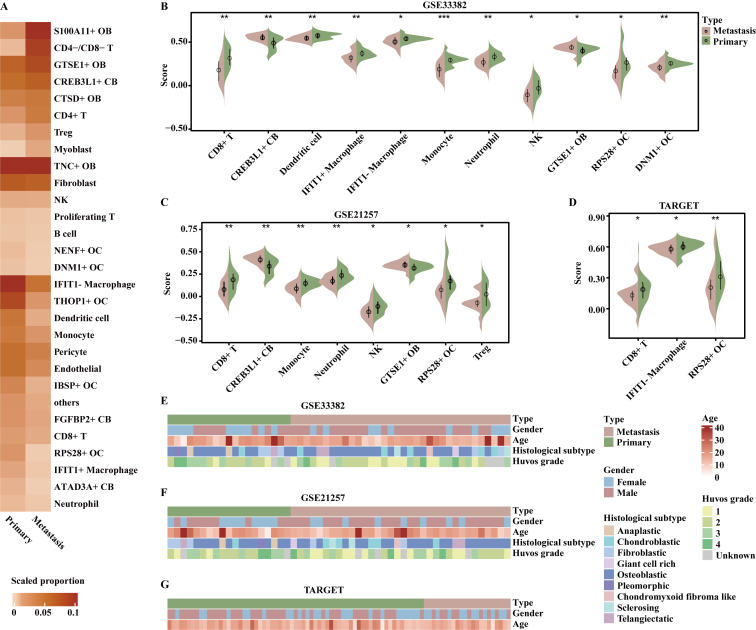
*GTSE1*^+^ OB cells and *CREB3L1*^+^ CB cells were enriched in metastatic tumors. **(A)** The proportions of cell subsets between primary and metastatic tissues were shown in the heatmap, and the color represented the scaled cellular proportions. **(B**–**D)** Significantly differential ssGSEA scores of cell-specific differential expression genes between primary and metastatic samples in GSE33382 (B), GSE21257 (C), and TARGET (D). **(E**–**G)** Heatmap of clinical information, including type, age, gender, histological subtype, and Huvos grade in GSE33382 (E), GSE21257 (F), and TARGET (G). *p* values were calculated by a two-tailed Wilcoxon rank-sum test. ∗*p* < 0.05, ∗∗*p* < 0.01, ∗∗∗*p* < 0.001.

The proportion of each subset was estimated in each sample and scaled across tissues (Fig. 2A). In short, two disparate patterns of cell abundance (primary OS high and metastatic OS high) were identified. Metastatic samples were enriched for *S100A11*^+^ OB cells, *GSTE1*^+^ OB cells, *CTSD*^+^ OB cells, CD4^−^/CD8^−^ T cells, *CREB3L1*^+^ CB cells, CD4^+^ T cells, Treg cells, and myoblasts, and contained fewer *IFIT1*^*−*^ macrophages, *THOP1*^+^ OC cells, dendritic cells, monocytes, CD8^+^ T cells, *DNM1*^+^ OC cells, *etc*., compared with primary samples (Fig. 2A).

Consistent with the results of scRNA-seq data, metastatic samples exhibited significantly increased scores of *GTSE1*^+^ OB cells and *CREB3L1*^+^ CB cells, while primary samples showed increased scores of CD8^+^ T cells, *IFIT1*^*−*^ macrophages, *RPS28*^+^ OC cells, monocytes, neutrophils, dendritic cells, *IFIT1*^+^ macrophages, and *DNM1*^+^ OC cells in bulk datasets (*p* < 0.05, two-tailed Wilcoxon rank-sum test; Fig. 2A–D). Although metastatic samples in bulk datasets were characterized by relatively low scores of NK cells (*p* < 0.05, two-tailed Wilcoxon rank-sum test; Fig. 2B, C), there was no difference in the proportion of NK cells between primary and metastatic samples in scRNA-seq data. Moreover, primary samples harbored increased scores of Treg cells in GSE21257, which was contrary to the results of scRNA-seq data (*p* < 0.05, two-tailed Wilcoxon rank-sum test; Fig. 2C). This might be due to the heterogeneity of datasets. Clinical information of bulk datasets showed no bias in proportions of OB and CB subtypes and age between metastatic and primary samples (Fig. 2E–G). In GSE21257 and GSE33382, females accounted for a higher proportion in metastatic samples than in primary samples (Fig. 2E–G).

### *GTSE1*^*+*^ OB cells are characterized by high proliferation activity

**Figure 3:**
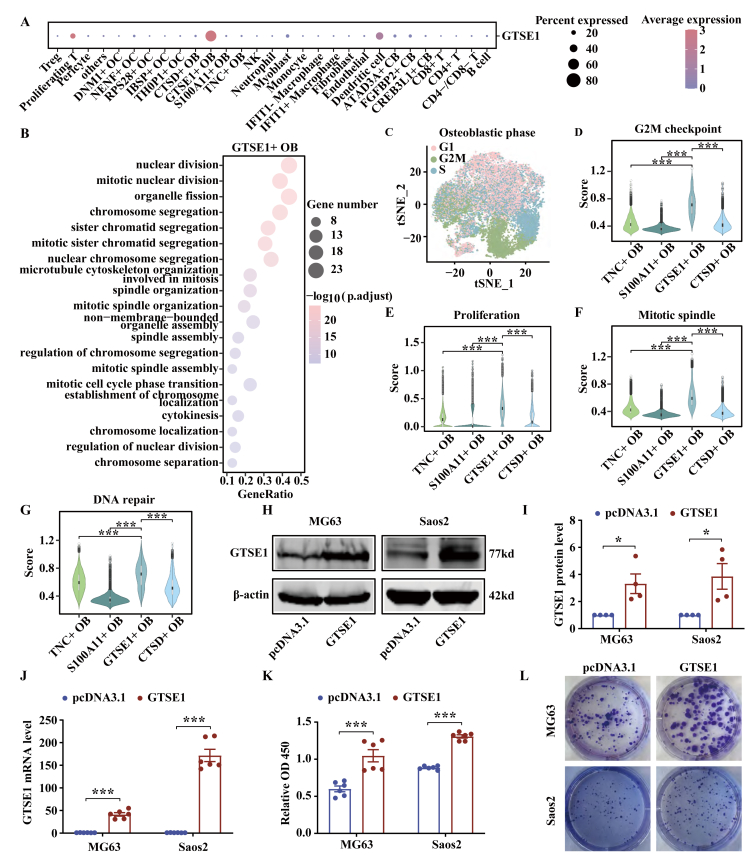
*GTSE1*^+^ OB cells were characterized by high proliferation activity. **(A)** The dot plots showing the proportions of cells and the average gene expression of *GTSE1* in all subsets. The dot sizes indicated the proportions of cells expressing the genes in each subset and the color spectrum represented the mean expression levels of *GTSE1*. **(B)** GO biological processes pathway enrichment analysis on differential expression genes of *GTSE1*^+^ OB cells. **(C)** t-SNE visualization of OB cells with each color representing a different stage of the cell cycle. **(D**–**G)** G2M checkpoint scores (D), proliferation scores (E), mitotic spindle scores (F), and DNA repair scores (G) among *TNC*^+^ OB cells, *S100A11*^+^ OB cells, *GTSE1*^+^ OB cells, and *CTSD*^+^ OB cells. **(H**–**J)** The transfection efficiency of *GTSE1* plasmid was confirmed by western blotting (H, I; *n* = 4) and quantitative real-time PCR (J; *n* = 6). **(K)** Cell viability was examined by CCK-8 assay (*n* = 6). **(L)** The images of colony formation were shown (*n* = 4). Data were shown as mean ± standard deviation of three independent cultures. *p* value was calculated by a two-tailed Wilcoxon rank-sum test in (D–G). *p* values were calculated by two-tailed student's *t*-test in (I–K). *p* value was calculated by the hypergeometric test in (B). ∗*p* < 0.05, ∗∗∗*p* < 0.001.

*GTSE1* showed the highest expression in OB cells, but its expression in stromal cells was low or undetectable (Fig. 3A). *GTSE1* expressed in *GSTE1*^+^ OB cells of OS subtypes with different histopathological patterns and clinical behaviors with no bias (Fig. S1D, E). The *GSTE1*^+^ OB cells were characterized by high expression of genes involved in nuclear division, mitotic nuclear division, and chromosome segregation pathways (false discovery rate < 0.05, hypergeometric test; Fig. 3B). *GTSE1*^+^ OB cells, which were mainly distributed in G2, M, or S phase of the cell cycle, harbored enhanced activities of G2M checkpoint, proliferation, mitotic spindle, and DNA repair pathways (*p* < 0.001, two-tailed Wilcoxon rank-sum test; Fig. 3C–G). The human OB cell lines MG63 and Saos2 were used to overexpress *GTSE1*. Protein and mRNA levels of GTSE1 were increased in *GTSE1*-transfected MG63 and Saos2 cells, which were assessed by western blotting and quantitative real-time PCR assays (*p* < 0.05, two-tailed student's *t*-test; Fig. 3H–J). As shown in Figure 3K and L, overexpression of *GTSE1* significantly increased cell viability and promoted growth through CCK-8 and colony-formation assays (*p* < 0.05, two-tailed student's *t*-test).

**Figure 4:**
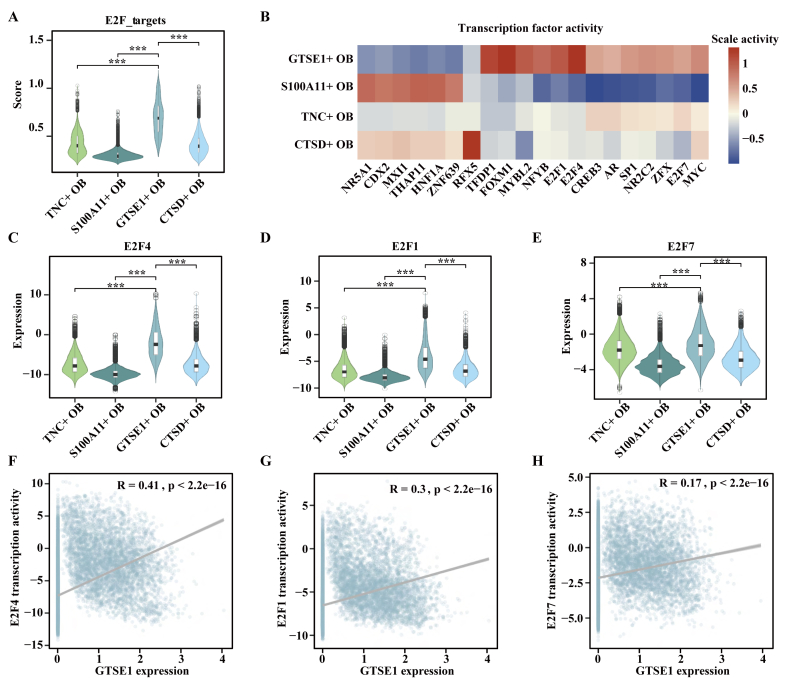
E2F target pathway was activated in *GTSE1*^+^ OB cells. **(A)** The ssGSEA scores of E2F target pathway among *TNC*^+^ OB cells, *S100A11*^+^ OB cells, *GTSE1*^+^ OB cells, and *CTSD*^+^ OB cells. **(B)** The top 20 transcription factors in OB cells. Dark red color represented higher activities of transcription factors, while dark blue color indicated lower activities. **(C**–**E)** Expression value of *E2F4* (C), *E2F1* (D), and *E2F7* (E) among *TNC*^+^ OB cells, *S100A11*^+^ OB cells, *GTSE1*^+^ OB cells, and *CTSD*^+^ OB cells. **(F–H)** Correlations between *GTSE1* expression value and activities of *E2F4* (F), *E2F1* (G), and *E2F7* (H). *p* value was calculated by a two-tailed Wilcoxon rank-sum test (A and C–E) and Spearman rank correlation analysis (F–H). ∗∗∗*p* < 0.001.

E2F target pathway was activated in *GSTE1*^+^ OB cells (*p* < 0.001, two-tailed Wilcoxon rank-sum test; Fig. 4A). Consistent with enrichment analysis, we observed a high activity score for *E2F4*, *E2F1*, and *E2F7* in *GSTE1*^+^ OB cells (Fig. 4B). The members of E2F family except *E2F6*, showed the highest expression in *GTSE1*^+^ OB cells among OB cells (*p* < 0.001, two-tailed Wilcoxon rank-sum test; Fig. 4C–E; Figs. S2A–D). In addition, the expression level of *GTSE1* was positively correlated with the activities of E2F family genes in OB cells, especially *E2F4* and *E2F1* (Fig. 4F, H; Figs. S2E–H).

### Up-regulation of *GTSE1* promotes the migration and invasion of OB cells

**Figure 5:**
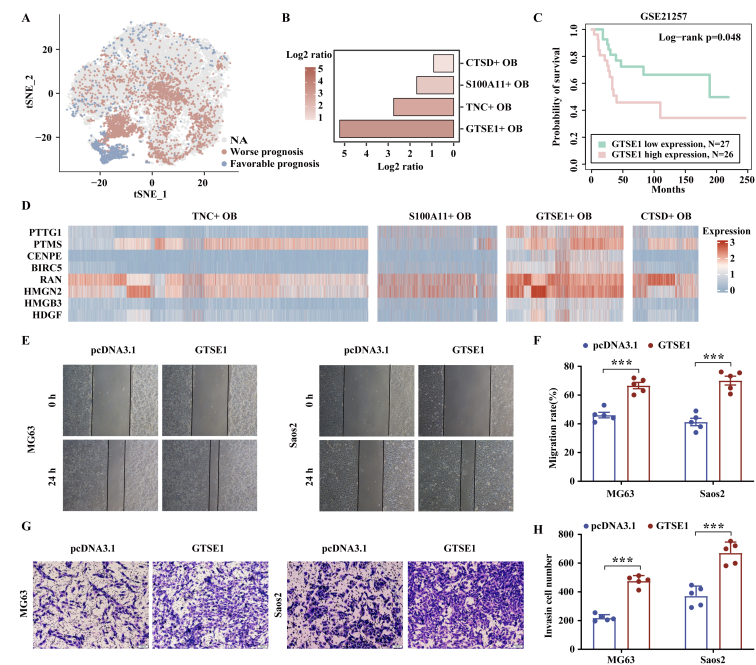
Up-regulation of *GTSE1* promoted the growth, migration, and invasion of OB cells. **(A)** Single-cell identification of cells with bulk sample phenotype correlation analysis using Scissor. The t-SNE visualization of the Scissor selected cells in OB cells. The red and blue dots were Scissor^+^ (worse prognosis) and Scissor^−^ (favorable prognosis) cells respectively. **(B)** The bar chart showing the ratio of the number of Scissor^+^ cells to the number of Scissor^−^ cells. A log2 ratio >0 meant a positive relation with worse survival. **(C)** The Kaplan–Meier overall survival curves of patients with OS stratified by *GTSE1* expression. **(D)** The heatmap showing the expression of stem cell markers in OB cells. **(E**–**H)** Wound healing (E, F; original magnification, ×40; *n* = 5) and transwell assays (G, H; scale bar = 100 μm; *n* = 5) showed that the abilities of migration and invasion of OB cells were increased after *GTSE1* overexpression. Data were shown as mean ± standard deviation of three independent cultures. *p* value was calculated by log-rank test in (C). *p* values were calculated by two-tailed student's *t*-test in (F, H). ∗∗∗*p* < 0.001.

Scissor analysis identified a total of 3,366 OB cells associated with the worse prognosis and 927 OB cells associated with the favorable prognosis (Fig. 5A). OS patients with enriched OB cells, particularly *GSTE1*^+^ OB cells, showed poor survival (Fig. 5B). *GSTE1* could serve as a prognostic marker of OS, whose high expression is associated with worse prognosis in GSE21257 (*p* = 0.048, log-rank test; Fig. 5C). All stem cell markers were widely highly expressed in *GTSE1*^+^ OB cells (Fig. 5D), suggesting that *GTSE1*^+^ OB cells may possess high stemness.^24^ The wound healing and transwell assays indicated that overexpression of *GTSE1* promoted migration and invasion in both MG63 and Saos2 cells (*p* < 0.05, two-tailed student's *t*-test; Fig. 5E–H).

### *GTSE1* depletion suppresses the growth, migration, and invasion of OB cells

**Figure 6:**
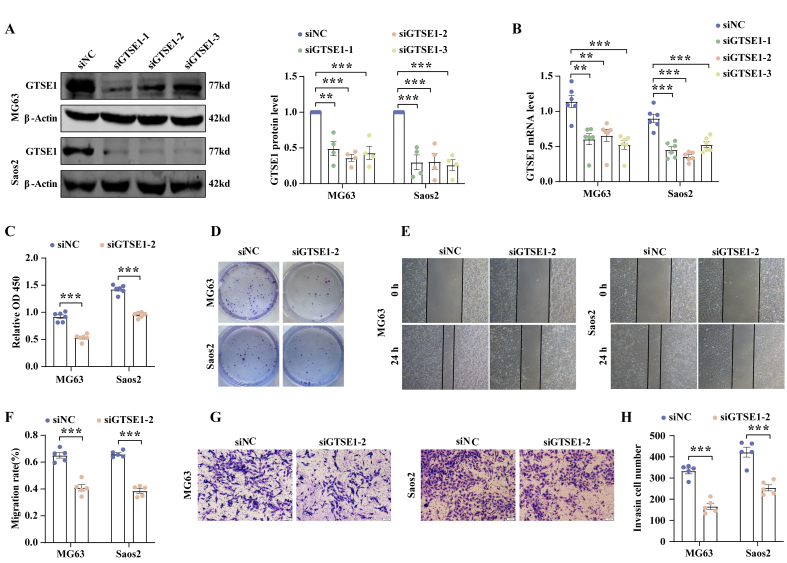
*GTSE1* depletion significantly suppressed the growth, migration, and invasion of OB cells. **(A, B)** Knockdown efficiency of *GTSE1* siRNAs was confirmed by western blotting (A; *n* = 4) and quantitative real-time PCR (B; *n* = 6). **(C)** Cell viability was examined by CCK-8 assay (*n* = 6). **(D)** Colony formation images (*n* = 4). **(E**–**H)** Wound healing (E, F; original magnification, ×40; *n* = 5) and transwell assays (G, H; scale bar = 100 μm; *n* = 5) showed that the abilities of migration and invasion of OB cells were decreased after *GTSE1* depletion. Data were shown as mean ± standard deviation of three independent cultures. *p* values were calculated by a two-tailed student's *t*-test. ∗∗*p* < 0.01, ∗∗∗*p* < 0.001.

We sought to assess the effect of *GTSE1* loss in OB cells on tumor growth and metastasis *in vitro*. *GTSE1* was depleted via three independent siRNAs in MG63 and Saos2 cells, and the efficiency of *GTSE1* depletion was successfully validated using western blotting and quantitative real-time PCR assays. A significant decrease in *GTSE1* expression was observed in cells with *GTSE1* depletion compared with the corresponding negative control (*p* < 0.05, two-tailed student's *t*-test; Fig. 6A, B). We chose siRNA-2 for the follow-up experiments. The CCK-8 and colony formation assays showed that *GTSE1* depletion inhibited the cell viability and growth ability in MG63 and Saos2 cells (*p* < 0.01, two-tailed student's *t*-test; Fig. 6C, D). *GTSE1* depletion not only impeded the cell migration (*p* < 0.01, two-tailed student's *t*-test; Fig. 6E, F) but also attenuated the ability of invasion (*p* < 0.05, two-tailed student's *t*-test; Fig. 6G, H) by wound healing and transwell assays.

### *GTSE1*^+^ OB cell and monocyte abundances are negatively correlated

**Figure 7:**
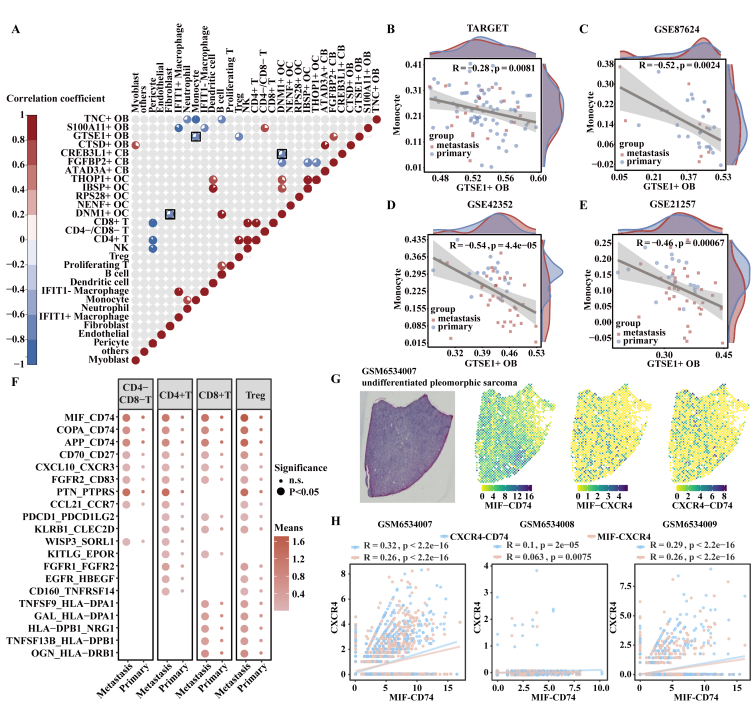
*GTSE1*^+^ OB cells were negatively correlated with monocyte abundances, while *CREB3L1*^+^ CB cells displayed increased interactions with fibroblasts in metastasis. **(A)** Pie charts represented the proportions of cell subsets with positive (*p* < 0.05 and correlation coefficient >0, Spearman rank correlation analysis, in red), negative (*p* < 0.05 and correlation coefficient <0, Spearman rank correlation analysis, in blue), or non-significant (*p* > 0.05, Spearman rank correlation analysis, in gray) correlations in GSE152048. The black boxes were correlations of interested cells. **(B**–**E)** Spearman rank correlation analysis between scores of differential expression genes of monocytes and *GTSE1*^+^ OB cells. The red density graphic showed the distribution of ssGSEA scores in metastatic samples, while the blue density graphic showed the distribution in primary samples, and each dot represented one sample. **(F)** The differential ligand–receptor cross-talk between *GTSE1*^+^ OB cells and T cell subsets (CD4^−^/CD8^−^ T cells, CD4^+^ T cells, CD8^+^ T cells, and Treg cells) in metastasis. The *x*-axis showed the tissues, while the *y*-axis showed the ligand–receptor pairs. The circle size represented the significance, while the color represented the mean expression of ligand and receptor. **(G)** Images of hematoxylin-eosin staining (left) for one sample and spatial colocalization of two genes (right). **(H)** Co-expression between colocalization of *MIF*-*CD74* and *CXCR4*-*MIF*/*CD74* pairs in three samples derived from GSE212526 (Pearson correlation analysis).

Negative correlations between the proportions of *GTSE1*^+^ OB cells and monocytes as well as *CREB3L1*^+^ CB cells and *DNM1*^+^ OC cells were observed in OS samples, but no correlation between the proportions of *GTSE1*^+^ OB cells and *CREB3L1*^+^ CB cells (Fig. 7A). We observed a negative correlation between the proportions of *GTSE1*^+^ OB cells and monocytes in four independent OS cohorts (Fig. 7B–E). Monocyte-specific DEGs were involved in immune response-related pathways, such as the T cell-mediated immunity and T cell-mediated cytotoxicity pathways (false discovery rate < 0.05, hypergeometric test; Fig. S3A). GO enrichment analysis showed that highly expressed genes in *GTSE1*^+^ OB cells and T cell subsets (CD4^−^/CD8^−^ T cells, CD4^+^ T cells, CD8^+^ T cells, and Treg cells) within metastatic samples were enriched in regulation of mononuclear cell migration pathway (false discovery rate < 0.05, hypergeometric test; Fig. S3C, D). The intercellular ligand–receptor analysis showed *GTSE1*^+^ OB cells and T cell subsets possessed preferential interactions of *MIF*-*CD74* and *COPA*-*CD74* pairs in metastatic OS (Fig. 7F). Furthermore, CellChat revealed that the number and strength of signaling pairs between *GTSE1*^+^ OB cells and CD8^+^ T cells increased in metastatic OS (Fig. S4A). *GTSE1*^+^ OB cells interacted with CD8^+^ T cells through the *MIF*-(*CD74*-*CXCR4*) pair in metastatic OS using the two methods (Fig. 7F; Fig. S4B). Bulk RNA transcriptome showed a higher positive correlation between *CD74* and *CXCR4* in metastatic than primary samples (Fig. S4C). Spatial transcriptomic data showed that co-expression levels of *MIF*-*CD74* and *CXCR4*-*MIF*/*CD74* were positively correlated in undifferentiated pleomorphic sarcoma, whereas the correlation was low in leiomyosarcoma (Fig. 7G, H; Fig. S4D). The above results indicated that the signaling interactions among *GTSE1*^+^ OB cells, monocytes, and T cells might contribute to a pro-metastatic microenvironment of OS. Among T cells, CD8^+^ T cells consistently showed lower infiltration in metastasis than primary samples in scRNA-seq and bulk datasets (Fig. 2A–D). To further illustrate the correlation among *GTSE1*^+^ OB cells, monocytes, and CD8^+^ T cells, we constructed the correlation network of three cell-specific DEGs (Fig. S4E). Intricate regulatory relationships were observed among them, with correlations existing between *GTSE1*^+^ OB cells and monocytes, *GTSE1*^+^ OB cells and CD8^+^ T cells, as well as monocytes and CD8^+^ T cells (Fig. S4E). *CEBPD*, *PTPRC*, *SERPINA1*, and *TUBA1A* were identified as hub genes in the regulatory network (Fig. S3B).

### *CREB3L1*^+^ CB cells display increased interactions with fibroblasts in metastasis

**Figure 8:**
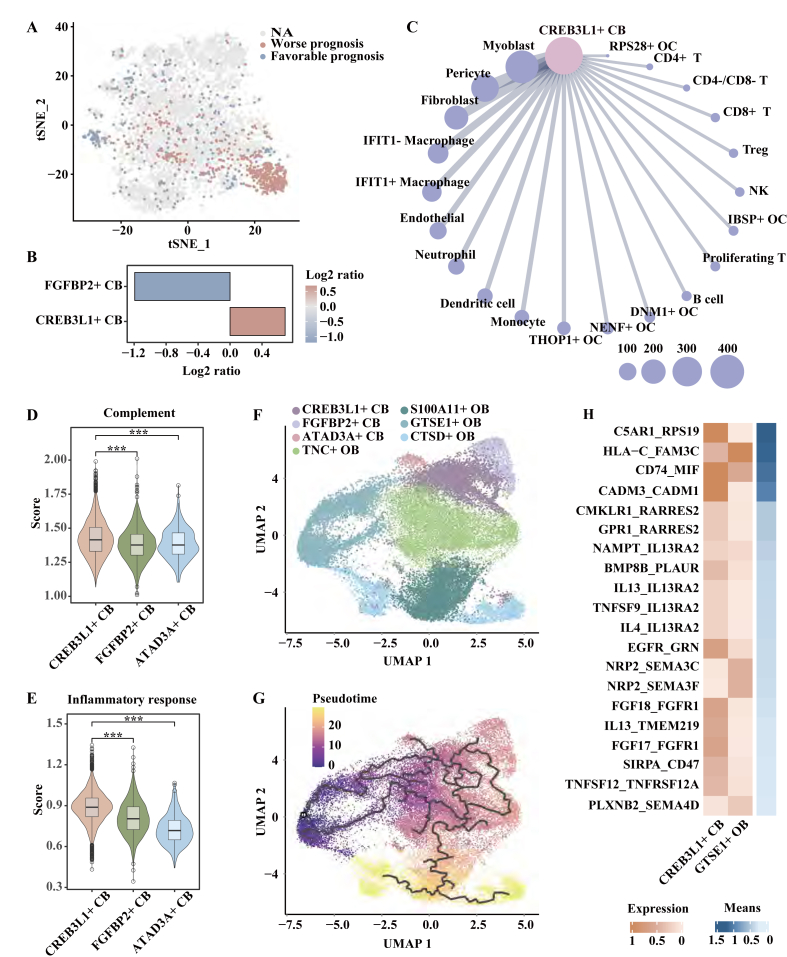
*CREB3L1*^+^ CB cells displayed increased interactions with fibroblasts in metastasis. **(A)** Single-cell identification of cells with bulk sample phenotype correlation analysis using Scissor. The t-SNE visualization of the Scissor selected cells in CB cells. The red and blue dots are Scissor^+^ (worse prognosis) and Scissor^−^ (favorable prognosis) cells, respectively. **(B)** The bar chart showing the ratio of the number of Scissor^+^ cells to the number of Scissor^−^ cells. A log2 ratio> 0 meant a positive relation with worse survival. **(C)** The ranked differential cross-talk between *CREB3L1*^+^ CB cells and non-malignant cells (metastatic samples versus primary samples) was indicated. Dot sizes represented the number of ligand–receptor pairs, which met the following requirements: *p* < 0.05 in metastatic samples and *p* > 0.05 in primary samples. The pink circle represented *GTSE1*^+^ OB cells, while the blue circle represented non-malignant cells. **(D, E)** Complement scores (D) and inflammatory response scores (E) among *CREB3L1*^+^ CB cells, *FGFBP2*^+^ CB cells, and *ATAD3A*^+^ CB cells. **(F, G)** Pseudotime trajectories of cancer cells generated using Monocle3. (F) The UMAP visualization of cancer cells. (G) Cells were color-coded for their corresponding pseudotime. **(H)** A heatmap of differential ligand–receptor cross-talk between *GTSE1*^+^ OB cells and *CREB3L1*^+^ CB cells (metastatic samples versus primary samples). The red color represented the expression value in cells. The blue color represented interaction scores, whereas the dark blue color indicated greater predicted interactions. Ligand–receptor pairs were listed along the left axis. *p* values were calculated by a two-tailed Wilcoxon rank-sum test (D–E). ∗∗∗*p* < 0.001.

*CREB3L1* was highly expressed in CB cells, especially in *CREB3L1*^+^ CB cells (Fig. S5C). Patients with high expression of *CREB3L1* exhibited worse overall survival in OS (*p* = 0.002, log-rank test; Fig. S5E). Scissor analysis identified that 356 *ATAD3A*^+^ CB cells were associated with a worse prognosis, but no *ATAD3A*^+^ CB cell was associated with a favorable prognosis (Fig. 8A). Patients enriched for *CREB3L1*^+^ CB cells and *ATAD3A*^+^ CB cells showed poor survival (Fig. 8A, B).

*CREB3L1*^+^ CB cell-specific DEGs not only participated in the extracellular matrix-related pathways but also involved in collagen-related pathways, such as extracellular matrix organization, extracellular structure organization, and collagen biosynthetic process pathways (false discovery rate < 0.05, hypergeometric test; Fig. S5A). These observations were consistent with up-regulated genes of metastatic *CREB3L1*^+^ CB cells compared with primary *CREB3L1*^+^ CB cells (false discovery rate < 0.05, hypergeometric test; Fig. S5B). Inferring of intercellular communication revealed that fibroblasts ranked high among all differential cross-talk cell types in metastasis (Fig. 8C), highlighting frequent interactions between *CREB3L1*^+^ CB cells and fibroblasts. Furthermore, *CREB3L1*^+^ CB cells were enriched with inflammatory response and complement pathways (Fig. 8D, E) and showed high expression of *S100A4* (*p* < 0.001, two-tailed Wilcoxon rank-sum test; Fig. S5F).

The proportion of *CREB3L1*^+^ CB cells negatively correlated with the proportion of *DNM1*^+^ OC cells, and so did *DNM1*^+^ OC cells with fibroblasts (Fig. 7A). Meanwhile, *CREB3L1*^+^ CB cells frequently interacted with fibroblasts (Fig. 8C). Correlation analysis showed intricate regulatory relationships among *CREB3L1*^+^ CB cells, *DNM1*^+^ OC cells, and fibroblasts (Fig. S5G). *PRSS35*, *FGFR1*, and *DNM1*, known to have a profound impact on metastasis,^25^, ^26^, ^27^ were identified as hub genes in the regulatory network (Fig. S5H).

Trajectory and pseudotime analysis showed that *GTSE1*^+^ OB cells were the origin of cancer cells, and *CTSD*^+^ OB cells were the end of cancer cells during the developmental trajectory (Fig. 8F, G). Inferring of intercellular communication between *GTSE1*^+^ OB cells and *CREB3L1*^+^ CB cells showed preferential interactions of *C5AR1*-*RPS19* and *CD74*-*MIF* in metastasis samples (Fig. 8H). *CPE*, a risk gene with high expression in OS and associated with distant metastasis,^28^ was identified as a hub gene in the cell-specific DEG correlation network of *GTSE1*^+^ OB cells and *CREB3L1*^+^ CB cells (Fig. S5D).

## Discussion

In this study, we investigated the heterogeneity between metastatic and primary OS using single-cell and bulk gene expression datasets. We unveiled increased *GTSE1*^+^ OB and *CREB3L1*^+^ CB cell proportions in metastatic OS. *GTSE1*^+^ OB cells, monocytes, CD8^+^ T cells, *CREB3L1*^+^ CB cells, and fibroblasts jointly coordinated the formation of pro-metastatic tumor microenvironment. *GTSE1* overexpression in OB cells contributed to OB cell invasion and metastasis, which may be a novel promoter for metastasis of OS.

In this study, we found that *GTSE1*^+^ OB cells and *CREB3L1*^+^ CB cells enriched in metastatic OS at the single-cell resolution. In bulk datasets, *GTSE1*^+^ OB cells and *CREB3L1*^+^ CB cells were marked by high infiltration in metastatic samples, suggesting that *GTSE1*^+^ OB cells and *CREB3L1*^+^ CB cells are two metastasis-enrich cancer cells. Owing to the intratumoral heterogeneity of OS and the presence of various subtypes (*e.g.*, OB cells, CB cells, and fibroblasts),^29^^,^^30^ we analyzed the expression of *GTSE1* across different clusters and subtypes of OS. *GTSE1* showed the highest expression in *GSTE1*^+^ OB cells, but no bias in different subtypes of histopathological patterns and clinical behavior. Emerging studies showed that *GTSE1* was up-regulated in patients with OS and correlated with poor survival involving DNA damage repair.^31^ Members of the E2F family, which were overexpressed in *GTSE1*^+^ OB cells, play important roles in DNA repair and cancer metastasis.^32^^,^^33^ *GTSE1*^+^ OB cells possessed high activities of G2M checkpoint and DNA repair pathways, suggesting that members of the E2F family promote DNA damage repair and G2/M transition in *GTSE1*^+^ OB cells (Fig. S6). However, the impact of *GTSE1* up-regulation on metastasis remained poorly understood. In this study, *in vitro* experiments showed that *GTSE1* overexpression accelerated the proliferation, migration, and invasion of MG63 and Saos2 cells. In contrast, silencing *GTSE1* impaired the proliferation, migration, and invasion of MG63 and Saos2 cells, suggesting that *GTSE1* overexpression may be a risk factor for metastasis in OB cells. Chemotherapy regimens, such as cisplatin, have not improved the 5-year survival rate of metastatic OS, primarily due to the acquired resistance of patients to chemotherapy.^31^ *In vivo* animal studies have demonstrated that GTSE1 knockdown enhances the therapeutic effect of cisplatin, resulting in inhibition of the growth of OS, whereas GTSE1 overexpression contributes to the resistance of OS to cisplatin.^31^ Exploring the role of *GTSE1* in patients of OS warrants our future work.

*GTSE1*^+^ OB cells develop an immune escape strategy by interacting with monocytes and CD8^+^ T cells. Signaling between cancer cells and non-malignant cells in the tumor microenvironment is crucial for tumor metastasis. CD8^+^ T cell immunity could block the metastasis.^34^ *GTSE1*^+^ OB cells and CD8^+^ T cells possessed preferential interactions of the *MIF*-(*CD74*-*CXCR4*) pair in metastatic OS. The *MIF*-*CD74* and *CXCR4*-*MIF*/*CD74* showed higher co-expression levels in undifferentiated pleomorphic sarcoma than in leiomyosarcoma. The *CD74*-*MIF* pair could promote cancer cell growth and metastasis.^35^ Meanwhile, MIF-mediated signaling via CD74 is dependent on receptor complex formation with CXCR4.^36^ Indeed, expression levels of CD74 and CXCR4 showed a high positive correlation in metastasis. These results revealed that *GTSE1*^+^ OB cells might occur immune escape from CD8^+^ T cell-mediated killing through the *MIF*-(*CD74*-*CXCR4*) interaction. Monocytes could shape T cells as the immunosuppressive or immunostimulatory phenotype,^37^ and monocytes enriched with genes involved in the T cell-mediated cytotoxicity pathway. Correlation analysis showed *GTSE1*^+^ OB cells and monocytes were negatively correlated both at single-cell and bulk RNA levels, suggesting that *GTSE1*^+^ OB cells might indirectly inhibit T cell cytotoxicity through monocytes. Monocytes might promote the expression of *GTSE1*^+^ OB cell-specific DEGs by up-regulating *CEBPD* and *SERPINA1*, two pro-metastatic genes.^38^^,^^39^

We revealed that *CREB3L1*^+^ CB cells acquired metastasis properties by activating extracellular matrix remodeling and inflammation. *CREB3L1*^+^ CB cells enriched with genes involved in the extracellular matrix organization pathway, suggesting that cancer cells could leverage extracellular matrix remodeling to create a microenvironment that facilitated metastasis.^40^ Frequent communication signals were observed between *CREB3L1*^+^ CB cells and fibroblasts. Fibroblasts could not only mediate extracellular matrix remodeling but also regulate inflammatory signaling pathways.^41^^,^^42^ Whether *CREB3L1*^+^ CB cells induced a pro-metastatic tumor microenvironment formation by secreting signals of inflammation and extracellular matrix remodeling to fibroblasts remains to be further researched. S100A4, which possesses pro-inflammatory and pro-metastatic activity,^43^ showed preferential expression in *CREB3L1*^+^ CB cells. The inflammatory response pathway was also activated in *CREB3L1*^+^ CB cells, which warrants further detail *in vitro* or *in vivo* biological experiments. Furthermore, ligand–receptor analysis showed preferential interactions of *C5AR1*-*RPS19* and *CD74*-*MIF* pairs between *GTSE1*^+^ OB cells and *CREB3L1*^+^ CB cells in metastasis, which could promote cancer cell growth and metastasis.^35^^,^^44^

There were some limitations in this study. While *GTSE1*^+^ OB cells and *CREB3L1*^+^ CB cells enriched in metastatic OS at single-cell and bulk RNA levels, it would be better to include other criteria such as protein data to further confirm this discovery. Although we have validated the role of *GTSE1* overexpression in OB cell lines, which promoted invasion and metastasis of OB cells, whether *GTSE1* overexpression promotes metastasis in patients with OS warrants further investigation. Some cross-talk signals between *GTSE1*^+^ OB cells and others (monocytes, CD8^+^ T cells, and *CREB3L1*^+^ CB cells) were involved in metastasis, which should be explored in our future work using spatial transcriptome data. Given the lack of a functional assessment of *CREB3L1*^+^ CB cells in metastatic OS, the present findings only revealed that *CREB3L1*^+^ CB cells were enriched in metastatic tumors. Future studies would require specific deletion of *CREB3L1* in CB cells to assess its role in promoting metastasis.

In conclusion, our finding reveals that *GTSE1*^+^ OB cells and *CREB3L1*^+^ CB cells enrich in metastatic OS, and a coordinated pro-metastatic tumor microenvironment driven by cancer cells and non-malignant cells. Enhancing the expression of *GTSE1* to promote OB cell invasion and metastasis, therefore, is a potential metastasis promoter.

## CRediT authorship contribution statement

**Linzhu Wang:** Investigation, Methodology, Writing – original draft. **Wenyue Li:** Investigation, Validation, Writing – original draft. **Weihang Ji:** Methodology, Validation. **Danyang Bing:** Methodology, Visualization. **Mingyue Liu:** Investigation. **Kaidong Liu:** Validation, Visualization. **Bo Chen:** Methodology, Software. **Zhangxiang Zhao:** Methodology, Supervision. **Yunyan Gu:** Conceptualization, Funding acquisition, Project administration. **Xuelian Li:** Conceptualization, Project administration. **Xiaoqiang E:** Conceptualization, Supervision. **Lei Yang:** Conceptualization, Funding acquisition.

## Data availability

The datasets analyzed during the current study are available from the public databases.

## Funding

This work was supported by the 10.13039/501100012166National Key Research and Development Program of China (No. 2023YFF1204600 to Lei Yang), the 10.13039/501100001809National Natural Science Foundation of China (No. 81972117 to Lei Yang; 32270710 to Yunyan Gu), the 10.13039/501100005046Natural Science Foundation of Heilongjiang Province, China (No. JQ2020H001 to Lei Yang), the Key R&D Program of Heilongjiang Province, China (No. GA23C002 to Lei Yang), the First Affiliated Hospital of Harbin Medical University Excellent Young Talents Funding, China (No. HYD2020JQ0013 to Lei Yang).

## Conflict of interests

The authors have no conflict of interests to declare.
