# Supplementary material for: GTSE1-expressed osteoblastic cells facilitate formation of pro-metastatic tumor microenvironment in osteosarcoma
Source: Genes Dis. 2025 Mar 7;12(6):101591. doi: 10.1016/j.gendis.2025.101591 (PMC12359159; doi:10.1016/j.gendis.2025.101591)
Supplement: Multimedia component 1 [file mmc1.docx]

**GTSE1-expressed osteoblastic cells facilitate formation of pro-metastatic tumor microenvironment in osteosarcoma**

**Supplementary Materials**

[Supplemental Methods 2](#_Toc179550432)

[Survival analysis and Scissor analysis 2](#_Toc179550433)

[Cell culture and transfection 2](#_Toc179550434)

[Quantitative real-time PCR 3](#_Toc179550435)

[Western blotting 3](#_Toc179550436)

[Correlation analysis 4](#_Toc179550437)

[Supplemental Table 4](#_Toc179550438)

[Supplemental Table 1. The osteosarcoma (OS) datasets used in this study 4](#_Toc179550439)

[Supplemental Figures 6](#_Toc179550440)

[Supplementary Figure 1. Percentage of subsets between primary and metastatic OS 6](#_Toc179550441)

[Supplementary Figure 2. Expression value of E2F family genes in OB cells 7](#_Toc179550442)

[Supplementary Figure 3. Interactions among *GTSE1*^+^ OB cells, monocytes and T cells 8](#_Toc179550443)

[Supplementary Figure 4. Communication between *GTSE1*^+^ OB cells and CD8^+^ T cells in metastasis was achieved through the *MIF*-(*CD74*-*CXCR4*) pair 9](#_Toc179550444)

[Supplementary Figure 5. The correlation network among *CREB3L1*^+^ CB cells, *DNM1*^+^ OC cells and fibroblasts 11](#_Toc179550445)

[Supplementary Figure 6. Members of the E2F family promoted cell proliferation in *GTSE1*^+^ OB cells 13](#_Toc179550446)

[Reference 13](#_Toc179550447)

# Supplemental Methods

## Survival analysis and Scissor analysis

Survival analysis was performed using “survival” package (v 3.3.1). Samples were stratified into two groups according to median expression value of *GTSE1* or *CREB3L1* in GSE21257. The Kaplan–Meier curve was plotted using R package “survival” and “survminer” (v 0.4.9).

R package “Scissor” (v 2.0.0) was applied to associate phenotypic data from GSE21257 with scRNA-seq data using overall survival (cox-regression) as dependent variable.^1^ The ratios of Scissor^+^ cells (positively associated with worse prognosis) number to Scissor^-^ cells (negatively associated) number were evaluated in each subset.

## Cell culture and transfection

The human OB cell lines MG63 (RRID: CVCL_0426) and Saos2 (RRID: CVCL_0548) were purchased from Wuhan Procell. All cell lines were authenticated using STR profiling within the last 3 years, and all the experiments were performed with mycoplasma-free cells. MG63 cell was cultured in MEM medium (Sigma, USA) supplemented with 10% fetal bovine serum (Biological Industries, Israel) and 1% (100 U/mL) penicillin and streptomycin (Beyotime Biotech, Jiangsu, China). Saos2 cell was cultured in McCOY’s 5A Medium (Meilunbio, Dalian, China) supplemented with 15% fetal bovine serum and 1% (100 U/mL) penicillin/streptomycin. All cells were maintained in an incubator at 37°C in an atmosphere containing 5% CO_2_. *GTSE1* plasmid (Genechem, Shanghai, China）and smart silencer (RiboBio, Guangzhou, China) were transfected using Lipofectamine 2000 (Invitrogen, Carlsbad, CA, USA) according to the manufacturer’s instructions. After transfection 6 h the medium was renewed by normal culture medium. The small interfering RNAs (siRNAs) sequence used are as follows:

*GTSE1* siRNA-1: GCCTACTCCTACAAATCAA

*GTSE1* siRNA-2: GCTGTAGGATCTGAAAGCA

*GTSE1* siRNA-3: GGGATGTTCTCCCTGACAA

## Quantitative real-time PCR

Total RNA was isolated using TRIzol reagent (Invitrogen, Carlsbad, CA, USA) according to the manufacturer’s protocol. cDNA reverse transcription kits (5 All-in-One RT Master Mix, TransGene Biotech, China) were used to reverse transcribe total RNA. The relative mRNA level was detected by SYBR Green on ABI 7500 Fast Real-time PCR system (Applied Biosystems, USA). β-actin was used as a normalized control. Data were analyzed by 2^-ΔΔCt^ method.

## Western blotting

The OB cell lines were lysed with RIPA buffer (Beyotime Jiangsu, China) supplemented with protease inhibitor on ice for 30 min, followed by centrifuging at 13,500 × rpm at 4°C for 15 min. The protein sample (60 µg) was separated on 10% SDS-polyacrylamide gel electrophoresis and transferred to pure nitrocellulose (Pall Life Sciences, Ann Arbor, MI, USA), then blocked with 5% fat-free dry milk for 1 h. The membranes were hybridized with β-actin (1:2000, Proteintech, 20536-1-AP) and *GTSE1* (1:500, Proteintech, 21319-1-AP) primary antibodies at 4°C overnight. Western blotting bands were imaged by Odyssey and quantified with Image Studio Ver 5.2 software.

## Correlation analysis

Spearman rank correlation analysis was used to calculate the correlations between *GTSE1* and transcription factors (TFs) belonging to E2F family in scRNA-seq data. The correlations between enrichment scores of two subsets were also calculated by Spearman rank correlation analysis. Moreover, the average expression values of genes in each sample were used for Spearman rank correlation analysis in scRNA-seq data. The correlations between expression levels of two genes were also calculated using Spearman rank correlation analysis in bulk datasets. *P* < 0.05 was recognized as a significant correlation. Cytoscape software (v 3.8.0, https://cytoscape.org/) was applied to visualize the correlation network of cell-specific DEGs.

# Supplemental Table

## Supplemental Table 1. The osteosarcoma (OS) datasets used in this study

| Data | Data Type | Metastasis sample | Primary  sample | Data Source |
| --- | --- | --- | --- | --- |
| GSE21257 | mRNA | 34 | 19 | http://www.ncbi.nlm.nih.gov/geo/ |
| GSE33382 | mRNA | 34 | 19 | http://www.ncbi.nlm.nih.gov/geo/ |
| GSE87624 | mRNA | 9 | 24 | http://www.ncbi.nlm.nih.gov/geo/ |
| TARGET | mRNA | 22 | 65 | https://ocg.cancer.gov/programs/target/ |
| GSE152048 | RNA single cell | 2 | 7 | http://www.ncbi.nlm.nih.gov/geo/ |

# Supplemental Figures

## Supplementary Figure 1. Percentage of subsets between primary and metastatic OS


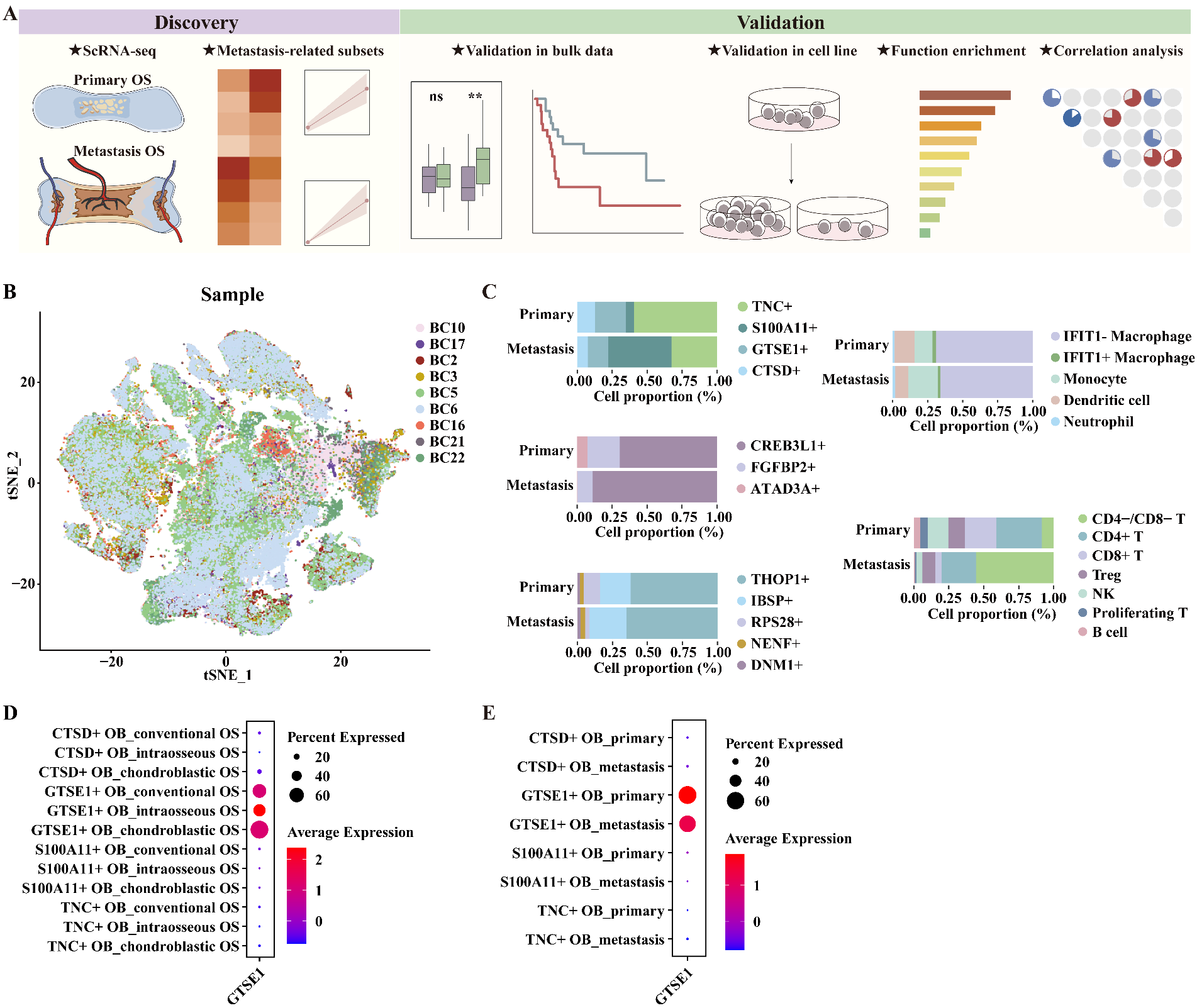


**Supplementary Figure 1. Percentage of subsets between primary and metastatic OS.** (A) The graphic overview for discovering and validating metastasis-enrich cells in OS. (B) t-SNE plot showing the distribution of samples, color-coded by samples. (C) Relative proportion of subsets of osteoblastic (OB) cells, chondroblastic (CB) cells, osteoclastic (OC) cells, myeloid cells and tumor-infiltrating lymphocytes (TILs). (D-E) Dot plots showing the proportions of cells and the average gene expression of *GTSE1* in all OB subsets.

## Supplementary Figure 2. Expression value of E2F family genes in OB cells


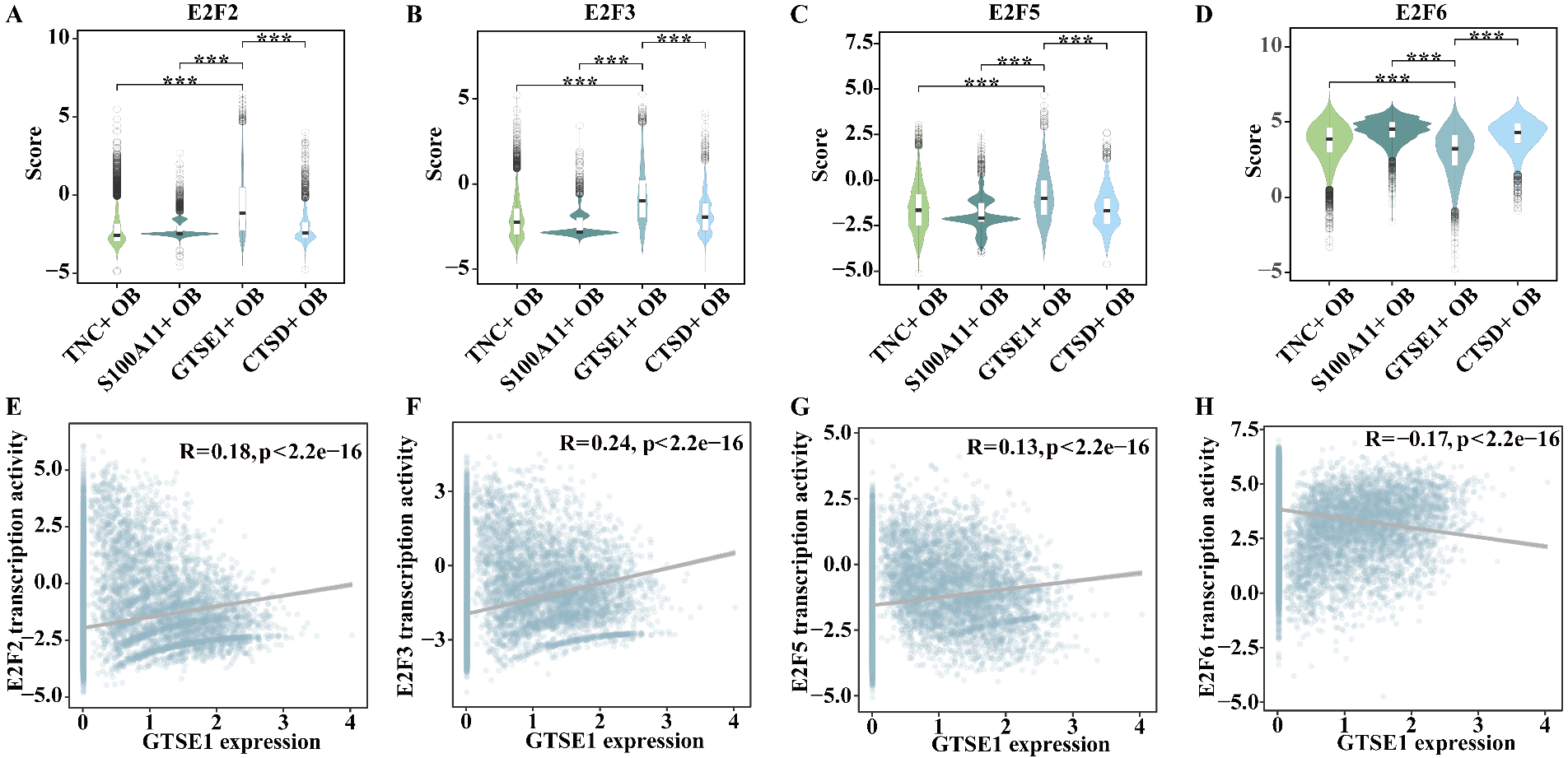


**Supplementary Figure 2.** **Expression value of E2F family genes in OB cells.** (A-D) Expression value of genes belong to E2F family among *TNC*^+^ OB cells, *S100A11*^+^ OB cells, *GTSE1*^+^ OB cells and *CTSD*^+^ OB cells. *P* values were calculated by two-tailed Wilcoxon rank-sum test. (E-H) Spearman rank correlation analysis between *GTSE1* expression value and activities of TFs belong to E2F family (Spearman rank correlation analysis). ****P* < 0.001.

## Supplementary Figure 3. Interactions among *GTSE1*^+^ OB cells, monocytes and T cells


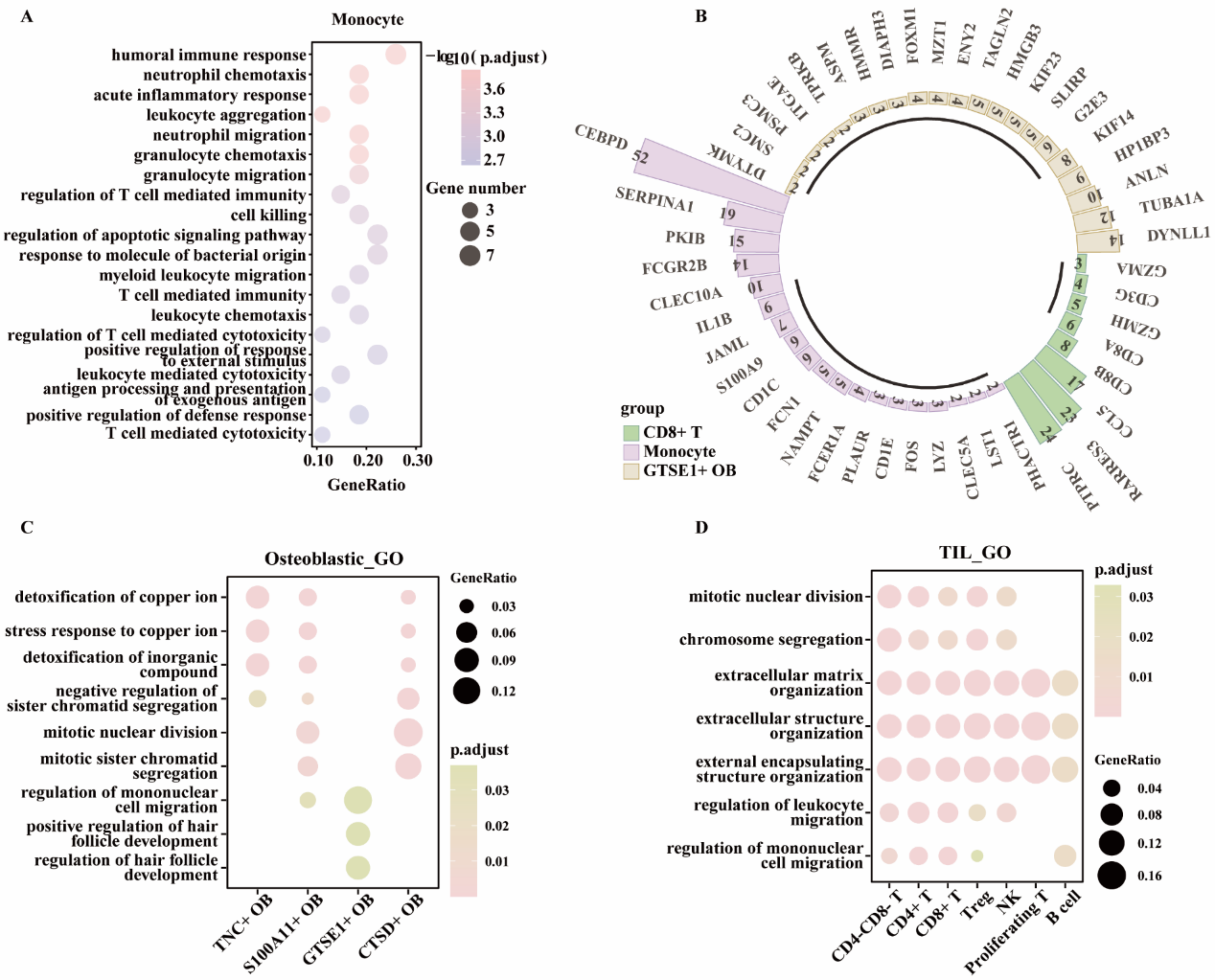


**Supplementary Figure 3. Interactions among *GTSE1*^+^ OB cells, monocytes and T cells.** (A) Gene Ontology (GO) biological processes pathway enrichment analyses on differential expression genes (DEGs) of monocytes. (B) The degree of nodes in the correlation network among *GTSE1*^+^ OB cells, monocytes and CD8^+^ T cells. (C-D) GO biological processes pathway enrichment analyses on highly expressed genes of OB subsets (C) and TIL subsets (D) within metastatic samples.

## Supplementary Figure 4. Communication between *GTSE1*^+^ OB cells and CD8^+^ T cells in metastasis was achieved through the *MIF*-(*CD74*-*CXCR4*) pair


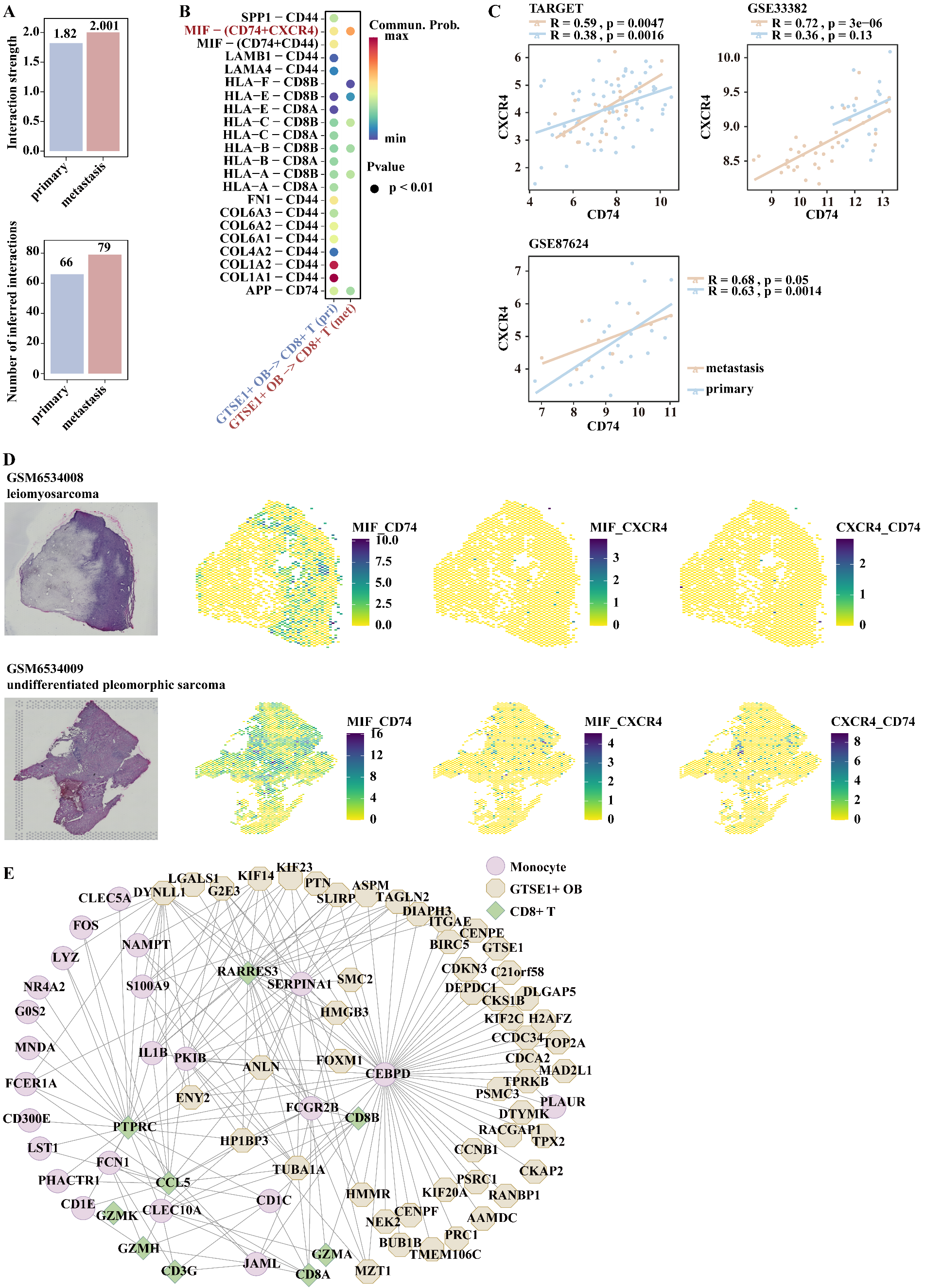


**Supplementary Figure 4.** **Communication between *GTSE1*^+^ OB cells and CD8^+^ T cells in metastasis was achieved through the *MIF*-(*CD74*-*CXCR4*) pair.** (A) Interaction strength and number of inferred interactions between *GTSE1*^+^ OB cells and CD8^+^ T cells. (B) Interacting ligand-receptor pairs from *GTSE1*^+^ OB cells to CD8^+^ T cells between primary and metastatic samples. (C) Correlations between expression levels of *CD74* and *CXCR4* in three bulk RNA transcriptome (Spearman rank correlation analysis). (D) Images of Hematoxylin-Eosin staining (left) for two samples and spatial colocalization of two genes (right). (E) The correlation network of cell-specific DEGs of *GTSE1*^+^ OB cells, monocytes, and CD8^+^ T cells. Red octagons represented DEGs of *GTSE1*^+^ OB cells, purple circles represented DEGs of monocytes, and green prisms represented DEGs of CD8^+^ T cells respectively (Spearman rank correlation analysis).

## Supplementary Figure 5. The correlation network among *CREB3L1*^+^ CB cells, *DNM1*^+^ OC cells and fibroblasts


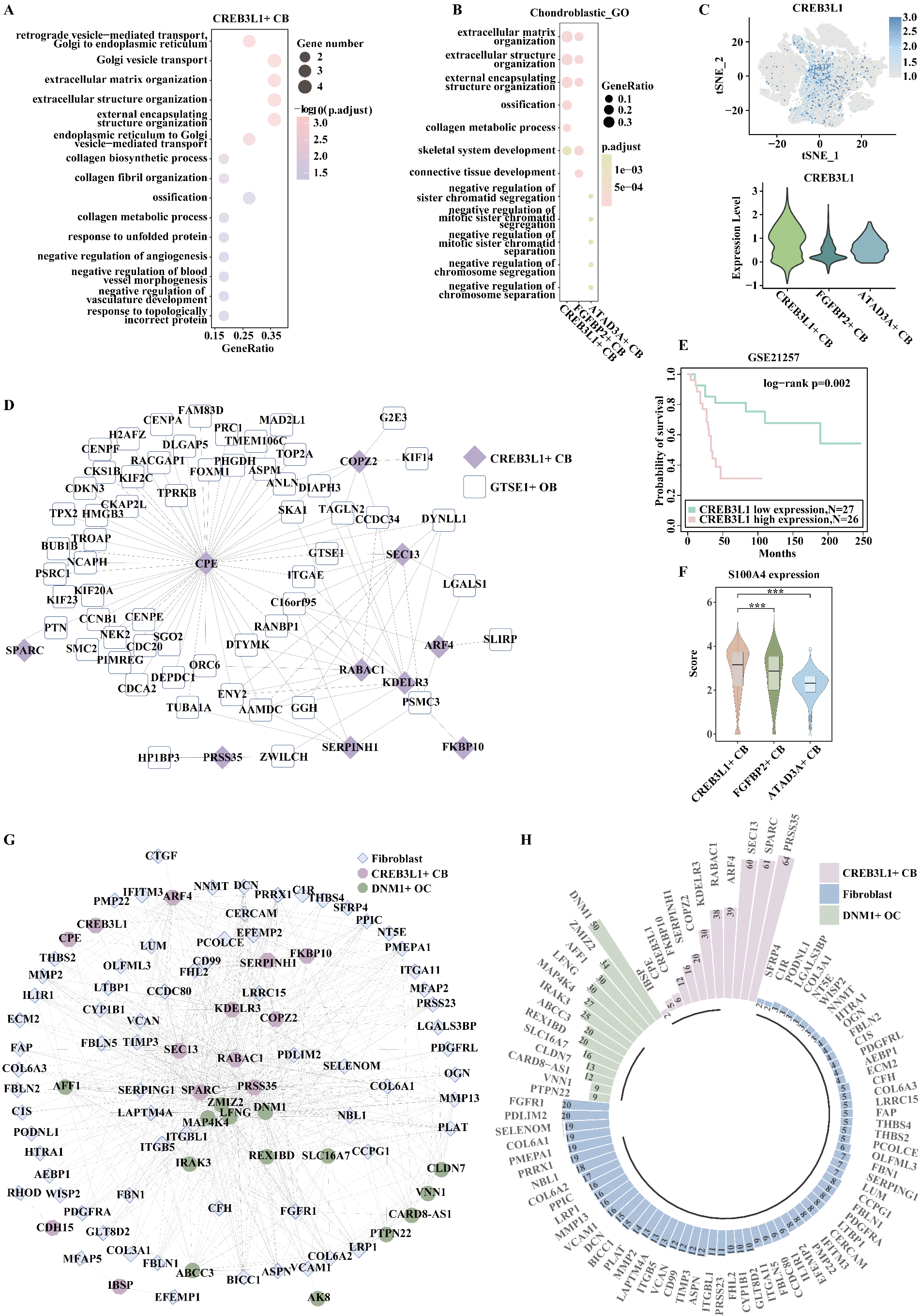


**Supplementary Figure 5. The correlation network among *CREB3L1*^+^ CB cells, *DNM1*^+^ OC cells and fibroblasts.** (A) GO biological processes pathway enrichment analyses on DEGs of *CREB3L1*^+^ CB cells. (B) GO biological processes pathway enrichment analyses on highly expressed genes of CB subsets within metastatic samples. (C) Expression value of *CREB3L1* among CB cells illustrated in t-SNE plot (up) and violin plot (down). (D) The correlation network of cell-specific DEGs of *CREB3L1*^+^ CB cells and *GTSE1*^+^ OB cells (Spearman rank correlation analysis). (E) The Kaplan–Meier overall survival curves of OS patients stratified by *CREB3L1* expression. (F) *S100A4* expression value among *CREB3L1*^+^ CB cells, *FGFBP2*^+^ CB cells and *ATAD3A*^+^ CB cells. (G) The correlation network of cell-specific DEGs of *CREB3L1*^+^ CB cells, fibroblasts and *DNM1*^+^ OC, and red octagons represented DEGs of *CREB3L1*^+^ CB cells, green circles represented DEGs of *DNM1*^+^ OC cells, blue prisms represented DEGs of fibroblasts respectively (Spearman rank correlation analysis). (H) The degree of nodes in the correlation network among *CREB3L1*^+^ CB cells, fibroblasts and *DNM1*^+^ OC cells. ****P* < 0.001.

## Supplementary Figure 6. Members of the E2F family promoted cell proliferation in *GTSE1*^+^ OB cells


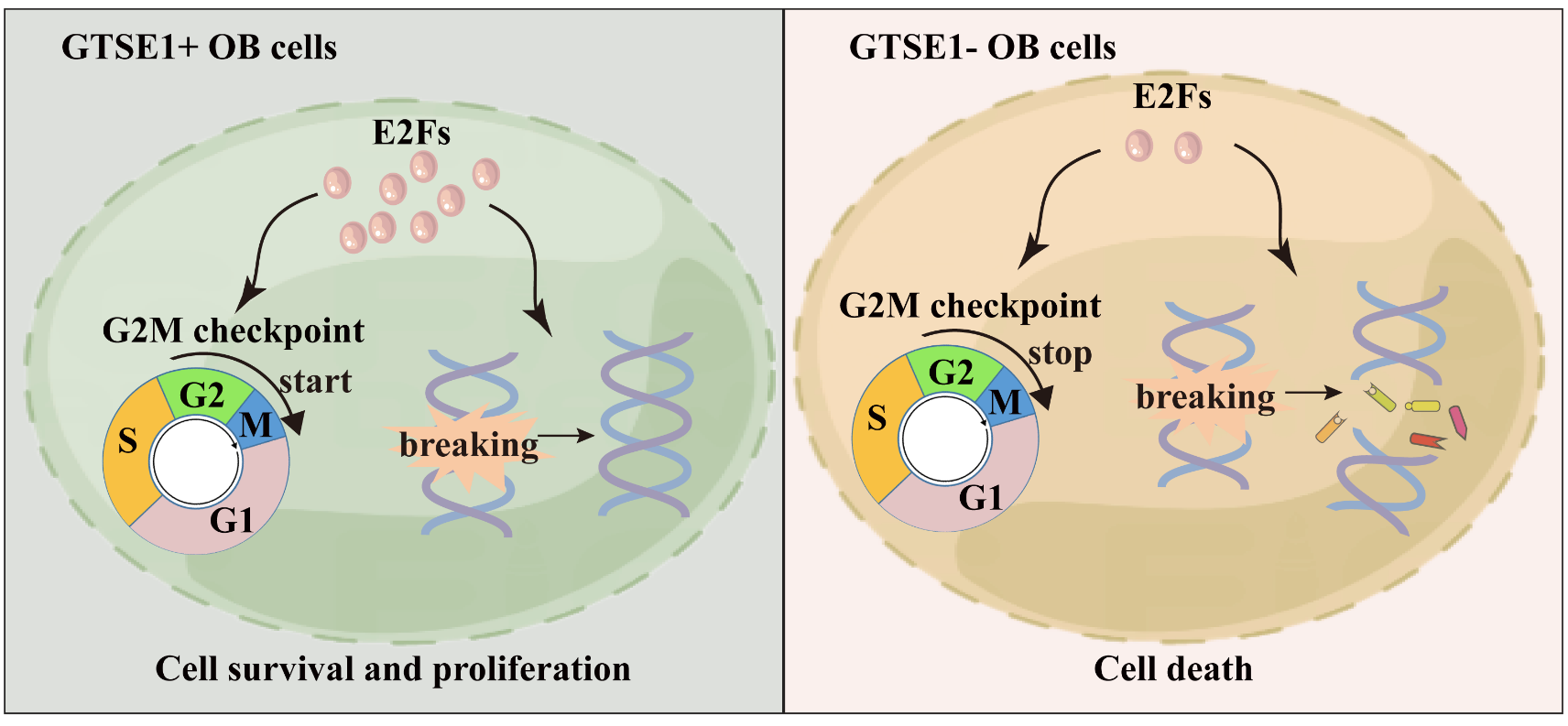


**Supplementary Figure 6. Members of the E2F family promoted cell proliferation in *GTSE1*^+^ OB cells.** Members of the E2F family were overexpressed in *GTSE1*^+^ OB cells. Members of the E2F family promoted G2/M transition and DNA damage repair in *GTSE1*^+^ OB cells.

# Reference

1. Sun D, Guan X, Moran AE, et al. Identifying phenotype-associated subpopulations by integrating bulk and single-cell sequencing data. *Nature biotechnology*. Apr 2022;40(4):527-538. doi:10.1038/s41587-021-01091-3
